# Controlled Release Film Forming Systems in Drug Delivery: The Potential for Efficient Drug Delivery

**DOI:** 10.3390/pharmaceutics11060290

**Published:** 2019-06-20

**Authors:** Thao T. D. Tran, Phuong H. L. Tran

**Affiliations:** 1 Department for Management of Science and Technology Development, Ton Duc Thang University, Ho Chi Minh City, Vietnam; trantruongdinhthao@tdt.edu.vn; 2 Faculty of Pharmacy, Ton Duc Thang University, Ho Chi Minh City, Vietnam; 3 School of Medicine, Deakin University, Geelong, Australia

**Keywords:** transdermal drug delivery, film-forming system, film, hydrogel, controlled drug release

## Abstract

Despite many available approaches for transdermal drug delivery, patient compliance and drug targeting at the desired concentration are still concerns for effective therapies. Precise and efficient film-forming systems provide great potential for controlling drug delivery through the skin with the combined advantages of films and hydrogels. The associated disadvantages of both systems (films and hydrogels) will be overcome in film-forming systems. Different strategies have been designed to control drug release through the skin, including changes to film-forming polymers, plasticizers, additives or even model drugs in formulations. In the current review, we aim to discuss the recent advances in film-forming systems to provide the principles and review the methods of these systems as applied to controlled drug release. Advances in the design of film-forming systems open a new generation of these systems.

## 1. Introduction

The development of controlled drug delivery systems has generated substantial interest in pharmaceutical science in recent years [1,2,3]. In particular, transdermal drug delivery has attracted researchers with multiple approaches because multiple dosing or insufficient drug delivery often results in low therapeutic effects [4,5,6,7,8]. Among these techniques, films (patches) and gels have been extensively designed for use in skin diseases or wound care in the past decades [9,10,11,12,13,14,15,16,17]. These dosage forms can also contain drugs for therapeutic applications.

Films or patches have the advantages of being a drug reservoir, having adhesive properties and providing precise performance at a targeted site on the skin, thereby prolonging drug release and improving therapeutic effects [18,19,20,21]. However, the fixed size and shape of these dosage forms are a limitation, especially for patients in some restrictive circumstances. In contrast to films, the hydrogel structure is flexible. However, the hydrogel structure has poor resistance to wearing and washing due to its hydrophilicity, although it has been reported in a sustained release form [22,23].

Fortunately, the ability of film-forming systems (FFSs) to transform non-solid dosage forms (such as gels and solutions) to films in situ has undergone substantial development to combine the advantages of both films and hydrogels. In fact, FFSs contain three main components—the drug, film-forming polymer and solvent(s) [24]. Upon contact with the target site (usually skin), the solvent will evaporate to form a film-loading drug [25].

The functionalization of polymers, drugs and other excipients in solid films could lead to controlled drug release. FFSs have shown therapeutic potential with various model drugs, especially in studying drug delivery through the skin. Here, we describe the basic concept of FFSs, including the generation of FFSs, and discuss the strategies used in developing a controlled drug release FFSs for effective therapies and the possible future development of this technology in the pharmaceutical industry.

## 2. Principles of Film-Forming Systems

**Figure 1:**
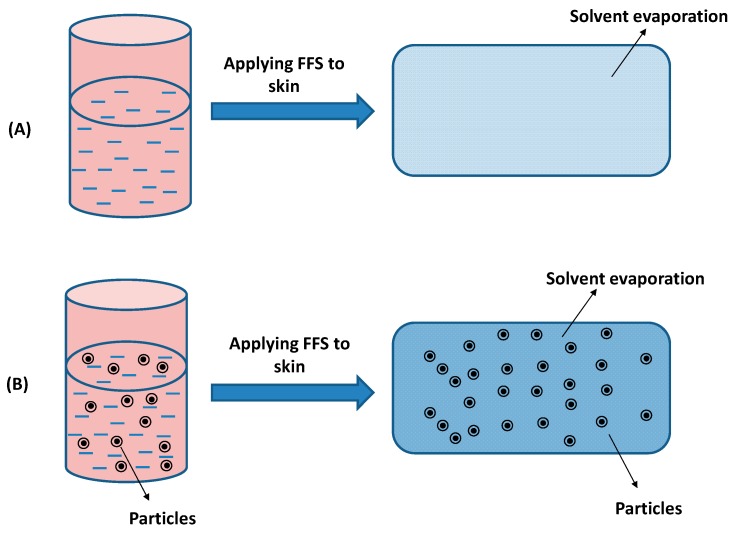
Illustration of film formation of film-forming systems (FFSs) containing solution (**A**) or dispersion of particles (**B**).

FFSs can be a solution or dispersion in which drug and film-forming excipients are dissolved/dispersed in a volatile solvent(s) [6,24]. The liquid state of the FFS depends on the solubility of drug/excipients or dispersions of encapsulated drug microparticles/nanoparticles in solvents. Upon contact with the skin, the solvents will evaporate and form a film with excipients. Figure 1 illustrates the film formation of FFSs. With solution FFSs, the polymer can have tight contact via molecular interactions to build an even film or smooth film [26]. In contrast, the coalescence of particles after solvent evaporation from dispersion FFSs can result in rough surface films. In both cases, a plasticiser is usually added to the FFS to reduce the brittleness of polymer films [27,28,29].

## 3. The Generation of Film-Forming Systems

### 3.1. Film-Forming Solutions

**Figure 2:**
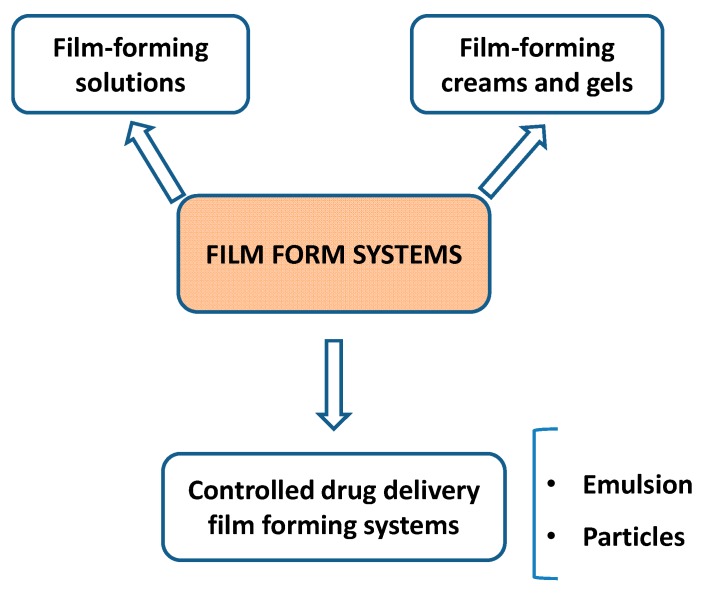
Illustrations of the generation of film-forming systems.

Since 1996, Amit Misra et al. has described film-forming solutions that have been known as the first generation of film-forming systems [30] (Figure 2). These authors noted that a film containing testosterone had efficient adherence to the skin by having sufficient tack. Consequently, high amounts of testosterone were diffused through the skin with biphasic of delivery the hormone, i.e., slow release rate after the initial burst release. In detail, the solution, which was applied to the skin surface, contains a polymer matrix of poly (vinyl alcohol) and poly (vinyl pyrrolidone) in isopropanol with a plasticizer (liquid paraffin) and a surfactant (Tween 20). These formulations were tested with in vitro skin permeation using Wistar rats. In later work, the authors successfully developed the experiment to evaluate the biphasic pharmacokinetics of testosterone in animal models (rat and monkey models) [31].

However, the above studies only discussed the efficiency of drug delivery in vitro and in vivo without focusing on the fabrication or evaluation of the film-forming systems. Therefore, Ines et al. first investigated polymer screening and characterization methods in 2007 to identify suitable formulations that were successfully tested in vitro (human epidermis) and in vivo (pigs) [32,33]. Important factors such as drying time, viscosity, cosmetic attractiveness, integrity, stickiness and mechanical properties were suggested [32]. In fact, quick drying time on the skin is necessary for patient compliance. Moreover, suitable viscosity ensures easy application of FFSs. The formulation also needs to be invisible for cosmetic attractiveness. The formulation should be non-sticky to avoid adhesion. Most importantly, FFSs must be retained for sustained drug release.

With the advantages of solutions, this type of film-forming system was recently developed and applied with spray formulations. For example, film-forming systems containing fluconazole and voriconazole were developed in 2009 and 2017, respectively [34,35]. The optimal formulation of fluconazole with Eudragit^®^ RS 100 and ethyl cellulose was a flexible and mucoadhesive film when sprayed on human skin [34]. Similarly, the mixture of Eudragit^®^ RLPO and ethyl cellulose showed that the spray formulation has the potential for applications in dermatological fungal infections due to the deep penetration of voriconazole into the skin [35].

Currently, most of the available market products are film-forming solutions, such as Axiron^®^, Lamisil Once^®^, Medspray^®^. In 2008, Acrux (Australia) initiated a phase III clinical trial of Axiron^®^ (the film-forming solution of testosterone) [36]. Axiron^®^ was approved by the US Food and Drug Administration in 2010 and licenced to Eli Lilly globally [36,37]. The application dose (30 mg testosterone) of Axiron^®^ comes in the form of a metered-dose pump (or twist) supplied with an applicator [38]. Another marketed film-forming solution is Lamisil Once^®^ (GlaxoSmithKline Consumer Healthcare Australia Pty Ltd.) which contains terbinafine hydrochloride for the treatment of athlete’s foot (tinea between the toes) with just one required application [39]. The ability of prolonged release in the skin of Lamisil Once^®^ is the advantage of this product for a single administration [24,40]. Medspray^®^ (MedPharm Ltd., UK) is the technology using “patch-in-a-can” concept which can also offer long residence of dosing in the skin or mucosal membrane after spraying film-forming solutions [41,42]. The “Liqui-Patch technology” technology (Epinamics GmbH, Germany) is also a sprayable film-forming solution [43]. The developed spray hood accompanied by the airless pump in this technology can maximize the film formation and minimize the cross-contamination [43].

### 3.2. Film-Forming Creams and Gels

Although film-forming solutions have proved to be effective formulations, researchers sought alternative dosage forms as a second generation of FFSs, such as creams or gels. The advantages of second-generation FFSs are high adhesiveness and easy application [6,44]. One noticeable report was the development of a cream containing the eutectic of lidocaine (7%) and tetracaine (7%) for application in cutaneous pulsed dye laser treatment [45]. This convenient system was then demonstrated to be effective for local anaesthesia in various studies and was approved by the Food and Drug Administration [46,47,48,49].

In 2003, Na-Mi et al. developed a film-forming soft hydrogel for transdermal delivery of testosterone using polyvinyl alcohol with a skin-permeation enhancer and an adhesive agent [50]. Polyisobutylene was used as an adhesive agent in the gel to reduce the contact angle and surface energy [50,51]. The gel was observed for 2–3 min for forming a film after application, and the thin film was retained over one day.

In another intensive effort, a bioadhesive film was made to improve the quality of FFSs [52]. The authors designed an organic-inorganic hybrid gel with a comfortable film (thin and flexible) and high cosmetic attractiveness (transparent) [52]. This research also indicated that this bioadhesive film could facilitate drug diffusion by decreasing crystalline parts of the PVA structure [52].

Film-forming gels have shown potential in applications for wound healing. One advantage of film-forming gels is easy application to the wound site compared to wound dressings, especially uneven wounds, such as curved or shaped wounds [14,53]. For example, Dong et al. showed that FFSs containing sodium fusidate significantly improved infection wound healing in vivo [44]. In that study, a gel component including PVP/PVA/propylene glycol/ethanol/water had a film-forming time of approximately 4 min with appropriate adhesiveness, flexibility and elasticity [44]. The optimal product also demonstrated high drug release and improved wound infection healing in rats compared to commercial products [44]. It has also been shown that an FFS containing chitosan-polyvinyl alcohol at a nanoscale could enhance antibacterial and antibiofilm properties for facilitating wound healing [54].

The noticeable commercial product of film-forming creams and gels is Durapeel technology (Crescita Therapeutics™, Canada). This technology has demonstrated its capability of sustained drug release up to 12 h into the skin and its application with various active ingredients [55].

### 3.3. Controlled Drug Delivery Film Forming Systems

With the advantage of creating in situ delivery, FFSs are feasible and promising in controlled drug release for topical drugs due to their ability to prolong drug release with an on-skin drug reservoir. The intensive development of FFSs in recent decades probably led to advances in controlled drug release. The inclusion of particles in the system would open a new generation of FFSs.

It has been shown that semi-solid emulsions in film-forming emulsions containing nonivamide could spread onto larger skin areas and sustain dermal release for chronic pruritus therapy [56]. Although the sustained release was attributed to the ratio of water soluble and insoluble polymers, these features of emulsions in FFSs became a focus of studies on reducing the frequency of the application of topical drugs. Later, the same research group demonstrated similar results with capsaicinoids for treatment of chronic pruritus with a constant permeation rate maintained for 12 h for efficient drug concentration on the skin [57]. Those studies clearly demonstrated the advantage of this system compared to conventional emulsions. In the latest research, nonivamide was studied for loading on silica particles for incorporation into FFSs [58]. This study indicated that an FFS containing nonivamid loaded on silica particles not only sustained drug delivery but also had advantages of not using irritating emulsifiers in the FFS compared to two previous studies [56,57,58]. The other potential designs of FFS formulations for controlled drug release are discussed below.

## 4. Formulation Design of Controlled Drug Release Film-Forming Systems

**Figure 3:**
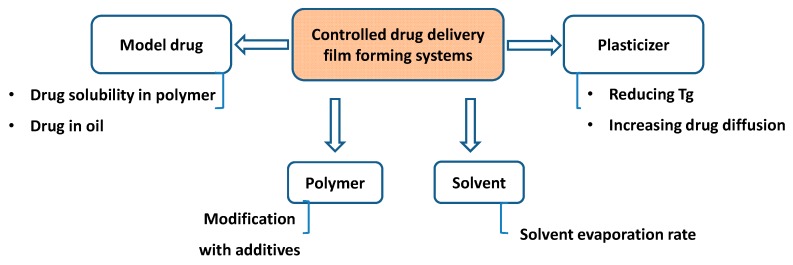
Illustration of factors in designing controlled drug delivery FFSs.

Generally, the formulations of FFSs contain a mixture of drug, excipients (polymers and plasticizers or additives such as penetration enhancers) and volatile solvents that can transform a liquid into a film on the skin surface after application. Therefore, several factors, such as the physicochemical properties of the drugs, the polymer and the plasticizer types and concentrations, the role of additives in formulations and the solvent evaporation will define the rate of drug release through the skin by modulation of drug saturation [33,59,60,61,62,63,64,65,66,67,68]. Therefore, these factors will be the focus of investigation for controlled drug release FFSs (Figure 3).

### 4.1. Model Drugs

The first step that should be considered in formulations is drug solubility in the film-forming polymers, which defines the drug saturation remaining on the skin surface. The ability of the drug to cross the skin greatly depends on the driving forces of drug saturation for triggering drug delivery. In recent research, drug solubility in the polymer has been suggested to be an important factor in controlling drugs crossing the skin [69]. In this study, methylphenidate was the free base and had a lower solubility in Eudragit^®^ RS than in Eudgragit^®^ E. Consequently, the higher degree of drug saturation resulted in higher drug release. Differential scanning calorimetry (DSC) has been proposed as a suitable drug solubility evaluation tool in the polymer for particular designs of FFSs for transdermal delivery [69]. Different weight fractions of drug and polymer were heated and measured by DSC. In fact, the melting enthalpy of drugs in different weight fractions of drug and polymer, which decreases during heating to the melting point, was used to measure drug solubility [69,70]. The maximum solubility of drugs was observed with the change in linearity of enthalpy because the drug could not be dissolved at this point [69].

**Figure 4:**
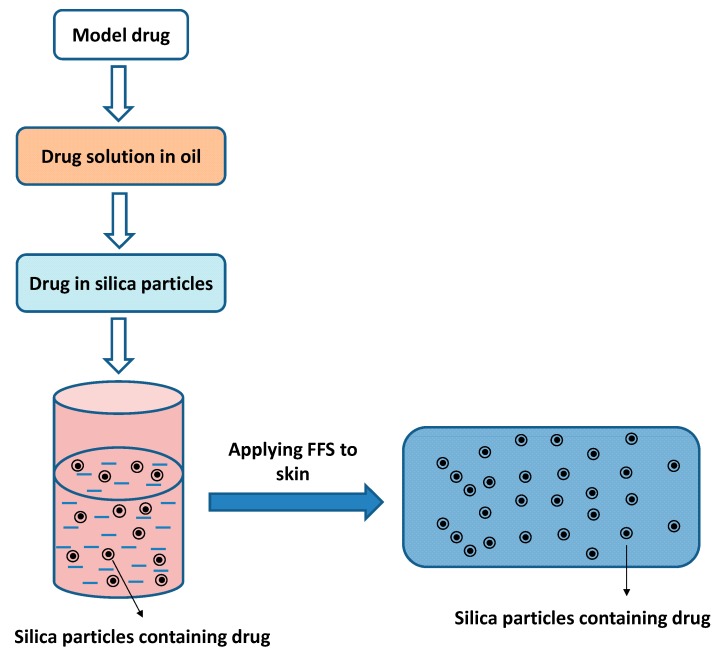
Illustration of a film-forming system containing particles for controlled drug release [58,71].

To sustain drug release, Rouven et al. suggested dissolving model drugs in refined castor oil and then loading the drugs on the silica particles before incorporating them into film-forming polymer solution [58,71] (Figure 4). The percentage of the drug in oil was 6%, and the ratio between the drug solution and silica particles was 3:2, which was mixed with the plasticized Eudragit^®^ RS 30D for creating the formulation containing 0.9% drug [71]. The results of ex vivo skin permeation, penetration and in vivo skin tolerability from these studies suggested prolonged drug release and no skin irritation for healthy volunteers [71].

### 4.2. Polymers

Another strategy that has been used to control drug release is a modification of the film-forming polymer as a functional excipient. Oh et al. successfully grafted oleic acid on chitosan to increase drug skin permeation [72]. The inhibition of drug crystallinity formation and modulation of polymeric globules on the film caused by these grafts led to the improvement in drug permeability [72]. In another study, Hazel et al. used lipidic medium-chain triglycerides in a hydrophobic polymer (Eudragit^®^ and Dermacryl^®^) or hydrophilic polymer (hydroxypropyl cellulose) for an FFS to investigate betamethasone-17-valerate uptake into the skin [73]. The study indicated that the presence of medium-chain triglycerides in the hydrophobic polymer films facilitated the enhancement of betamethasone-17-valerate uptake into the skin compared to the hydrophilic polymer, which was explained by the hydrogen bond interaction between the model drug and the hydrophobic polymer [73]. The following is a brief summary of popular polymers used in FFSs.

*Cellulose derivatives*: Hydroxypropyl cellulose and ethyl cellulose are cellulose derivatives frequently selected for FFS preparations. While hydroxypropyl cellulose can be dissolved and swell to form a hydrogel in water, ethyl cellulose is water-insoluble [74,75,76]. However, ethyl cellulose can dissolve in an organic solvent to produce good film-forming properties and stability [77,78,79]. Typically, ethyl cellulose is used as a film-coating agent for sustained release in pharmaceutical formulations [80]. The mechanical properties of ethyl cellulose film depend on the molecular weight and viscosity [81]. Due to insolubility, ethyl cellulose is often preferred combined with hydroxypropyl cellulose for controlled drug release [82,83,84,85]. Specifically, the drug release and water permeability of the films depend on hydroxypropyl cellulose concentration in the films (the higher hydroxypropyl cellulose concentration is, the more increased drug release and permeability are) [82,83,86]. Moreover, hydroxypropyl cellulose is utilized with other polymers, such as Carbopol 934, to improve drug reservoirs in FFS formulations [87,88]. In addition to the role of gelling agent, Carbopol 934 and hydroxypropyl cellulose at an appropriate ratio could improve the drug release significantly (at the ratio 1:5 as reported) [87].

*Polyvinyl pyrrolidone (PVP)*: The use of PVP facilitates a flexible selection of solvent for FFSs due to the solubility in both organic solvents and water of this polymer to produce good film-forming capacities [89,90,91,92]. Other advantages of PVP in FFSs are high hygroscopicity, good biocompatibility, and the ability to increase bioadhesive strength, which is probably explained by the formation of hydrogen bonds and Van der Walls’ forces [89,90]. Therefore, PVP was investigated in various studies on wound dressing applications [44,93,94,95,96,97,98]. In some cases, the copolymer of polyvinylpyrrolidone-vinyl acetate is used instead of PVP [32]. The incorporation of vinyl acetate could result in reduced hygroscopicity [99].

*Polyvinyl alcohol (PVA)*: Although PVA is well-known in vivo biocompatibility, the application of PVA in FFSs is concerning and restricted because of low hydrophilicity, rigid film generation and insufficient elasticity [100,101,102]. Therefore, PVA blends are recommended to improve the characteristics of PVA for use in FFSs, such as combining it with PVP [44]. The miscibility and good blending of PVP-PVA are attributed to the formation of hydrogen bonds between the –C=O groups (in pyrrolidone rings of PVP) and –OH group (of PVA), resulting in good mechanical properties, good biocompatibility for various biomedical applications [100,103,104]. However, a large amount of PVP could lead to an open network structure and hence, inducing the formation of weak hydrogels [100,105]. Therefore, a small amount of PVP in a PVP-PVA blend has been recommended from 0.5–5% for stable hydrogels [100,105]. Moreover, a stable hydrogel was reported with the incorporation of low molecular weight PVA in the PVP-PVA blend as compared to the high molecular weight PVA [100].

*Chitosan*: Chitosan is a natural polysaccharide derived from chitin [106]. Due to its good biocompatibility and specific biological activities, chitosan has been exploited in various applications in drug delivery and regenerative medicine [107]. Specifically, the mucoadhesive nature and the presence of available functional groups in its chemical structure are important properties of chitosan to enable its utilizations in biomedical applications [108]. Similar to PVP, chitosan is usually applied for fabrication of a wound dressing due to the advantages of its biological properties (e.g., accelerating wound-healing), especially the blend of chitosan and PVP with antibacterial activity [106,109,110]. Moreover, the swelling and mucoadhesive properties have generated interest in FFSs, particularly film-forming gels [32,59,110,111,112]. For example, hydroxypropyl chitosan in controlled release FFS is a successful formulation that could demonstrate a higher permeation and penetration into the human nails as compared to a commercial product [113]. With regard to solubility, chitosan can be dissolved in an acidic solution, although it is insoluble in water and organic solvents [114,115].

*Polymethacrylates*: Although a broad range of polymers have been investigated for FFSs, polymethacrylates have been commonly studied for FFSs, such as Eudragit^®^ RS (ammonio methacrylate copolymer type B), Eudragit^®^ NE 40D (poly(ethyl acrylate-co-methyl methacrylate) 2:1), Eudragit^®^ E 100 (poly(butyl methacrylate, (2-dimethylaminoethyl)methacrylate, methyl methacrylate) 1:2:1), Eudragit^®^ RL PO (Ammonio methacrylate copolymer), and Eudragit^®^ S 100 (poly(methacrylic acid, methyl methacrylate) 1:2) [33,56,59,98,116,117]. Polymethacrylates have excellent properties for FFSs with high flexibility, skin compatibility and controlled drug release [118].

### 4.3. Plasticizers

In addition to polymers, plasticizers also contribute to drug delivery through the skin by not only reducing the glass transition temperature (Tg) of the formed polymeric films but also increasing drug diffusion [59]. For example, Eudragit^®^ RS, with high Tg (approximately 65 °C), should be incorporated with a plasticizer for use on the skin surface (temperature approximately 32 °C) [59,119,120]. Plasticizers can increase the free volume and chain mobility of polymers, thereby improving drug release [59,121,122].

### 4.4. Solvents

Although solid excipients play key roles in FFS formulations, the solvent evaporation rate is also involved in drug permeation through the skin. Specifically, a study on Eudragit^®^ RL as a polymer using different model drugs (flurbiprofen, ibuprofen and ketoprofen) indicated that the initial burst drug release could be archived if the evaporation rate of the mixture of acetone and isopropyl alcohol in the FFS was slow [123].

With the development of hydrogel nanoparticles with many applications and the benefits of nanoparticles, there is also a great opportunity for the inclusion of other types of nanoparticle research in FFSs [124,125,126,127,128,129,130,131,132,133,134,135,136]. For example, a future study will provide a detailed understanding of how nanosized drugs can be incorporated and stabilized in FFSs and be delivered effectively to targeted sites. New insight into system mechanisms, such as the film transformation phenomenon and the ability of FFSs to deliver different drug nanoparticles, will be discovered. Therefore, knowledge of encapsulation and release behaviours of nanoparticles in FFSs will be improved to develop advanced materials for effective topical products.

## 5. Evaluation of the Physico-Chemical Properties of Controlled Drug Release Film-Forming Systems

### 5.1. Physicochemical Characterizations

The physical characterization of FFSs is associated with their hardness, adhesiveness, drying time, flexibility, and elasticity to maintain the contact between the film and skin as well as the resistance from the physical movement of skin [44,137]. The hardness and adhesiveness can be measured by a texture analyser [44,137]. While hardness is defined as the maximum force during a movement of a disc into a gel, adhesiveness is the ability of an FFS to adhere on a disc which is determined by the force of a disc in the upward movement [137]. To evaluate the in vitro film-forming drying time, a small amount of FFS is put into an acrylic plate (or Petri dish, microscopic slide) and observed by naked eyes [44,59,138,139]. The observations of the degree of uniformity, cosmetic appearance (such as transparency), and peelability are also evaluated in some circumstances [6,59]. The stickiness of an outer dried FFS can be determined by applying cotton wool with low pressure to evaluate the ability of adherence to fibres [32]. Furthermore, the mechanical properties of a dried FFS such as flexibility, elasticity are investigated through the determination of tensile strength and elongation at break using texture analysis [44]. The maximum weight which is loaded at the time of rupture of dried FFS is defined as the tensile strength [32,44]. Besides, the comparison of initial dried FFS length with FFS length at break determines the elongation at break (%) [32,44]. Microscopies such as atomic force microscopy and Raman microspectroscopy also provide the mechanical and topographical information of a dried FFS at the nanoscale [117]. Other common instrumental analyses are recommended for various purposes of a study. For instance, to detect drug crystallinity in a dried FFS, powder X-ray diffraction and differential scanning calorimetry are critical analyses; or to identify the molecular interactions between the drug and other excipients (or between excipients), Fourier-transformed infrared spectroscopy and nuclear magnetic resonance should be included [27,76,140,141,142].

### 5.2. Drug Release and Drug Penetration Evaluation

For an evaluation of drug release, Franz diffusion cell or dialysis instrument is selected for in vitro study [44,56]. However, the dialysis tube has a disadvantage of dissolving the dried FFS during its contact with the dissolution medium. In contrast, while the donor compartment of the Franz diffusion cell is the place to attach an FFS to a diffusion membrane (or excised skin for ex vivo study) and expose the dried FFS to the atmosphere, the receiver compartment contains the diffusion medium allowing drug permeation from a dried film [56]. Controlled drug release FFSs for a prolonged time can affect the passive diffusion of water through the skin, which may change the percutaneous drug absorption (e.g., change blood flow, skin temperature, etc.) [32,143,144,145]. Therefore, the determination of water vapour permeability of a dried film by the VapoMeter is an important factor in controlled drug release FFSs [32,73]. Additionally, to further investigate uptake of drug in the skin, ex vivo skin penetration should be performed. Typically, an FFS is applied to the intact skin surface of porcine ears evenly and maintained at 32 °C with pH 7.4 as the diffusion medium in Franz diffusion cells [73,146,147,148]. At a predetermined time post-application, the residual FFS is cleaned from the skin surface [73,149]. Subsequently, the stratum corneum, epidermis and dermis are removed by taping and stripping to extract the amount of drug penetration using an organic solvent [73,148,150]. The amount of drug in filtered extracts is determined by an appropriate method (e.g., high performance liquid chromatography) [148].

## 6. Conclusions

Several studies of FFSs have demonstrated potential applications in therapeutics. The efforts to design controlled drug release FFSs are expected to provide an efficient system for the modulation of drug release, not only for transdermal drug delivery but also for other types of drug delivery. The generation and inclusion of particles in FFSs can improve drug delivery at the targeted site. This strategy and many other systems (e.g., nanoparticles) will open the possibility of optimizing the manufacturing process for FFSs in the future. Moreover, other strategies including the optimization and modification of polymers, plasticizers, and drugs are also key factors in the research and development of controlled drug release FFSs.
